# Identification of characteristics and construction of nomogram to predict the survival probability of mesonephric carcinoma patients: A population‐based analysis and a case report

**DOI:** 10.1002/cnr2.1940

**Published:** 2023-11-29

**Authors:** Zhuoran Li, Dongyu Liu, Wenlong Wei, Zhisheng Huang, Yuzhen Mo, Haowei Huang

**Affiliations:** ^1^ Department of Radiotherapy Guangzhou Red Cross Hospital of Jinan University Guangzhou Guangdong China; ^2^ Department of Radiology Guangzhou Red Cross Hospital of Jinan University Guangzhou Guangdong China; ^3^ Department of Burns and Plastic Surgery Guangzhou Red Cross Hospital of Jinan University Guangzhou Guangdong China; ^4^ Department of Rehabilitation Guangzhou Hospital of Integrated Traditional Chinese and Western Medicine Guangzhou Guangdong China

**Keywords:** mesonephric carcinoma, nomogram, rare tumor, SEER stage, survival

## Abstract

**Background:**

Mesonephric carcinoma (MC) is a very rare tumor with less than 70 cases had been reported. The rarity of MC has restricted its research, resulting in the lack of published guidelines.

**Objective:**

To summarize the characteristics and construct an external‐validated nomogram to predict the survival of MC patients.

**Method:**

Sixty‐four qualified patients derived from the Surveillance, Epidemiology, and End Results Plus database, and one patient from the Guangzhou Red Cross Hospital were enrolled. The entire cohort was randomly divided into a development (70%) and a validation cohort (30%). The Kaplan–Meier method and univariate and multivariate Cox regression analyses were applied. Two nomograms were established to predict the 3‐to‐8‐year survival probability of MC patients, which were evaluated by *C*‐index, ROC curves, DCA curves, and calibration plots.

**Results:**

The average survival time of MC patients was 84.22 ± 50.66 months. No significant difference was shown among different groups of race, primary site, tumor differentiated grade, and FIGO stages, while different SEER stages did distinguish patients' survival time, which indicated that the SEER stage standards might be a better staging system in the MC patients than FIGO stage (*p* = .0835). Additional survival analyses showed that MC patients benefited from shorter waiting times to begin treatment, accepting surgery, regional lymph node examination, radiotherapy, and chemotherapy. Two nomograms were established, both of which got satisfied scores in *C*‐index, ROC curves, DCA curves, and calibration plots.

**Conclusion:**

Sufficient regional lymph nodes examined, and applying radiotherapy in high‐risk patients are recommended in MC patients. Nomograms established in the present study had good predicting and discriminating capabilities, which would be helpful in patients' individual risk estimation, management, counseling, and follow‐up.

## 1 INTRODUCTION

Mesonephric carcinoma (MC) is a very rare tumor deriving from remnants of the mesonephric duct, with only less than 70 cases reported up to date,^1^, ^2^, ^3^, ^4^, ^5^, ^6^, ^7^, ^8^, ^9^, ^10^, ^11^ which is a kind of non‐HPV‐related cervical adenocarcinoma, representing <1% of all cervical carcinomas, and can be clinically aggressive.^12^ MC usually occurs in the cervix, vagina, uterine body, and ovary.^13^, ^14^, ^15^, ^16^, ^17^ However, it can also occur in the bladder in male patients.^18^, ^19^ The pathological features of MC are the combination of multiple growth patterns, showing glandular, tubular, and papillary, which etiology and precursor lesions remain unclear. CD10, calretinin, GATA3, and TTF1 have been reported to be useful immunohistochemical markers for distinguishing MC from its morphologic mimics.^20^ Because of its broad morphologic spectrum and immunohistochemical profile, MC presents a diagnostic challenge to pathologists and should be differentiated from a variety of entities including clear cell adenocarcinoma (CCA), urothelial carcinoma with glandular differentiation, florid mesonephric hyperplasia, endometroid carcinoma, serous carcinoma, nephrogenic adenoma, and metastatic carcinoma.^21^ Due to the extremely low occurrence rate, the epidemiological and biological characteristics of MC still remain unclear. The rarity of MC has restricted its prospective studies, and as a result, there is no prospective research published so far with the optimal management strategy of MC patients remaining largely unknown.^22^

In this study, we reported a case of MC, as well as obtained the largest cohort of MC patients to date, and developed two nomogram models to predict the 3‐to‐8‐year survival probability of MC patients. As far as we know, this is the first attempt to construct such nomograms, which might provide valuable assistance in individual risk assessment, treatment decision‐making, follow‐up planning, and counseling for MC patients.

## 2 ETHICAL APPROVAL AND CONSENT TO PARTICIPATE

This study was approved by the Ethics Committee of Guangzhou Red Cross Hospital of Jinan University.

## 3 METHOD

### 3.1 Retrospective design, data sources, and population selection

The data for this study were obtained from the latest Surveillance, Epidemiology, and End Results (SEER) Plus database, specifically the SEER 8, 12, and 17 Registries with additional treatment information submitted in November 2021. Additionally, one female patient diagnosed with MC at the Guangzhou Red Cross Hospital of Jinan University was included in the study. The SEER database is a well‐regarded and comprehensive source of cancer‐related information, covering approximately 50% of the U.S. population.^23^ For this study, records of patients diagnosed with MC between 1990 and 2019 were extracted using the SEER*Stat software (version 8.4.1), with a minimum follow‐up period of 4 years.

MC was identified based on the International Classification of Diseases for Oncology third edition (ICD‐O‐3) code 9110, which encompasses subtypes 9110/0, 9110/1, and 9110/3. Male patients were excluded from the analysis due to the rarity of MC in males, with only four male patients identified in the SEER database with incomplete information.

**FIGURE 1:**
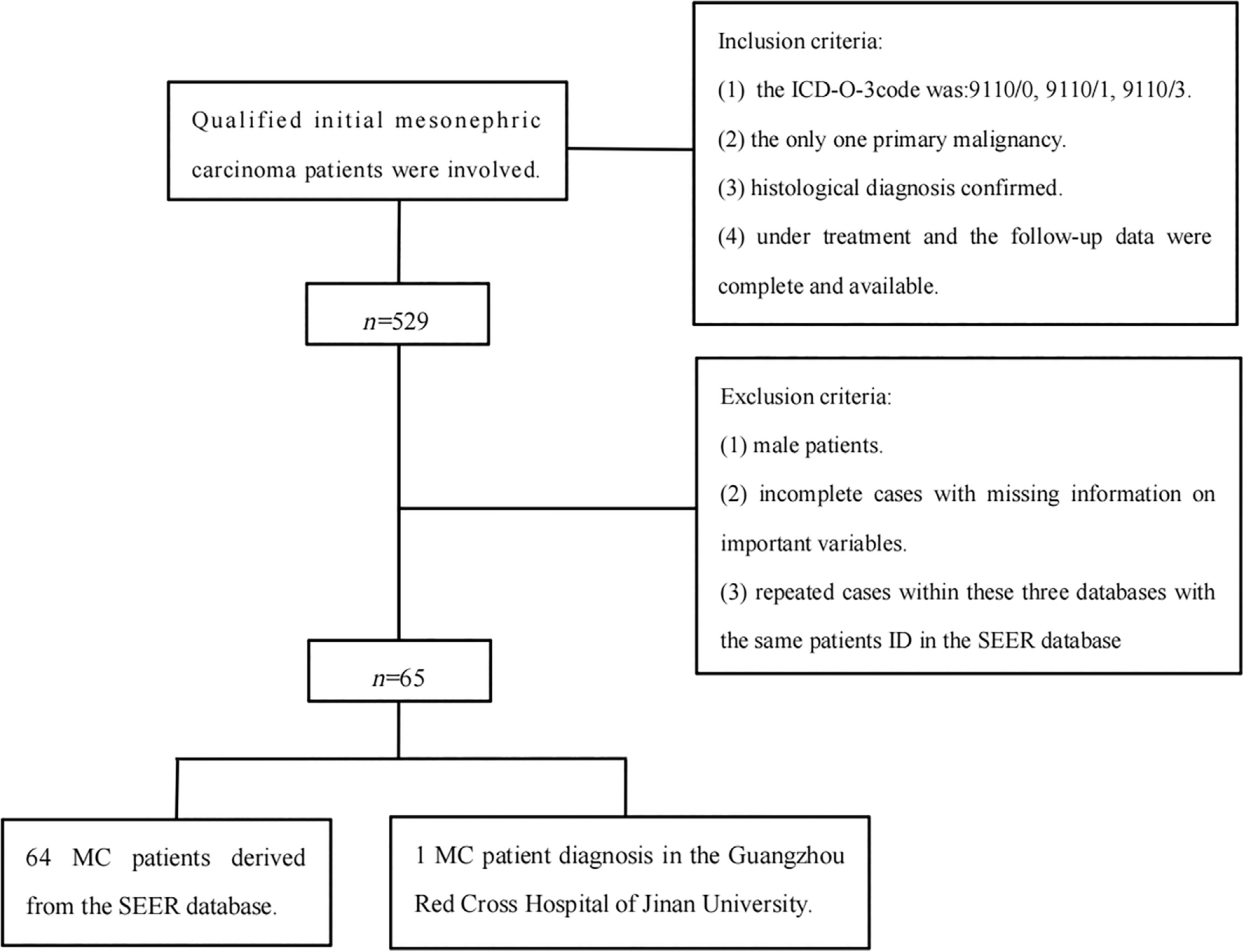
The study flowchart.

The inclusion criteria for the study were as follows: (1) MC as the primary malignancy; (2) confirmation of MC through histological diagnosis; (3) availability of complete follow‐up data during the course of treatment. On the other hand, the exclusion criteria were as follows: (1) male patients; (2) cases with missing information on important variables; (3) exclusion of repeated cases within the three databases with the same patient ID in the SEER database. The flowchart outlining the selection process of cases is provided in Figure 1.

### 3.2 Variable declaration and outcome

A total of 64 female MC patients from the SEER database and one patient from the Guangzhou Red Cross Hospital of Jinan University were included. Various variables were extracted for analysis, including age, race, year of diagnosis, primary site, tumor differentiation grade, TMN stage, surgery information, radiotherapy information, chemotherapy information, time from diagnosis to treatment, SEER stage, number of lymph nodes examined/removed/positive, tumor size, site of distant metastasis, International Federation of Gynecology and Obstetrics (FIGO) stage, survival status, cancer‐specific death, cause of death, and survival months.

Age was categorized into six groups: ≤40, 41–49, 50–59, 60–69, and 70+. Race was grouped as white, black, and other. Tumor differentiation grade was classified as grade I (well differentiated), grade II (moderately differentiated), grade III (poorly differentiated), and grade IV (undifferentiated or anaplastic). The primary site was divided into cervix uteri, corpus uteri, ovary, other female genital organs, kidney/renal pelvis/urinary bladder, and vagina.

The SEER stage was categorized into localized, regional, and distant based on the SEER database classification. The duration from diagnosis to treatment was classified as less than or more than 1 month. The number of regional lymph nodes examined was regrouped as none, ≤10, 11–20, and ≥21. Tumor size was categorized as ≤5 and >5 cm. Detailed FIGO stages were regrouped into FIGO I, FIGO II, FIGO III, and FIGO IV. Overall survival (OS) in this study referred to the time from the initial cancer diagnosis to cancer‐specific death.

### 3.3 Study design and methods

The entire cohort was randomly divided into a development cohort (70%) and a validation cohort (30%) for the development and validation of the predicting model nomogram. The patient from the Guangzhou Red Cross Hospital of Jinan University was included in the validation cohort. Standardized mean differences (SMDs) were used to assess distributional differences in the baseline variables between the development and validation cohorts.

### 3.4 The Kaplan–Meier method

The Kaplan–Meier method was constructed to estimate the difference in OS between the development cohort and the validation cohort. Meanwhile, the Kaplan–Meier method was also applied to estimate the difference in OS among different groups of those variables. To avoid bias, patients' age at the year of diagnosis was enrolled as the adjusted variable when analyzing those baseline characteristics such as race, primary site, tumor differentiated grade, the SEER stage, and the FIGO stage. Meanwhile, the SEER stage also served as the adjusted variable, along with age, when analyzing the treatment variables such as surgery/chemotherapy/radiotherapy performed and regional lymph node status.

### 3.5 Variable selection

The univariate and multivariate Cox regression analyses were applied to identify variables that significantly affected the MC‐specific survival.

### 3.6 Nomogram construction and evaluation

In order to help the clinicians predict the survival probability of MC patients, two nomograms were established. The first nomogram model 1 was established based on the variables selected by the univariate and multivariate Cox regression analyses. Considering distant metastasis has been included in the clinical stage, lung metastasis was excluded when constructing nomogram models. Another simplified nomogram model 2 was established based on several key variables that clinicians would pay attention to. Next, we identified low‐risk and high‐risk patients by calculating the dichotomous quantiles of total points of the nomogram in the cohort and compared the difference in their survival time using the Kaplan–Meier method. Validation of the nomogram was performed by calculating the concordance index (*C*‐index) and plotting calibration curves by a bootstrapping method with 1000 resamples. Furthermore, the receiver operating characteristic (ROC) curves were drawn to estimate the predictive value by calculating the area under the ROC curve (AUC). Meanwhile, the decision curve analyses (DCA) were conducted to show the clinical effectiveness of the nomogram model.

### 3.7 Statistical analyses

All the analyses were performed using R software (version 4.21, https://www.r-project.org/). The significance level is set as *p* < .1 in the Kaplan–Meier analyses and *p* < .05 in univariate and multivariate Cox regression analyses.

## 4 RESULTS

### 4.1 Case report

**FIGURE 2:**
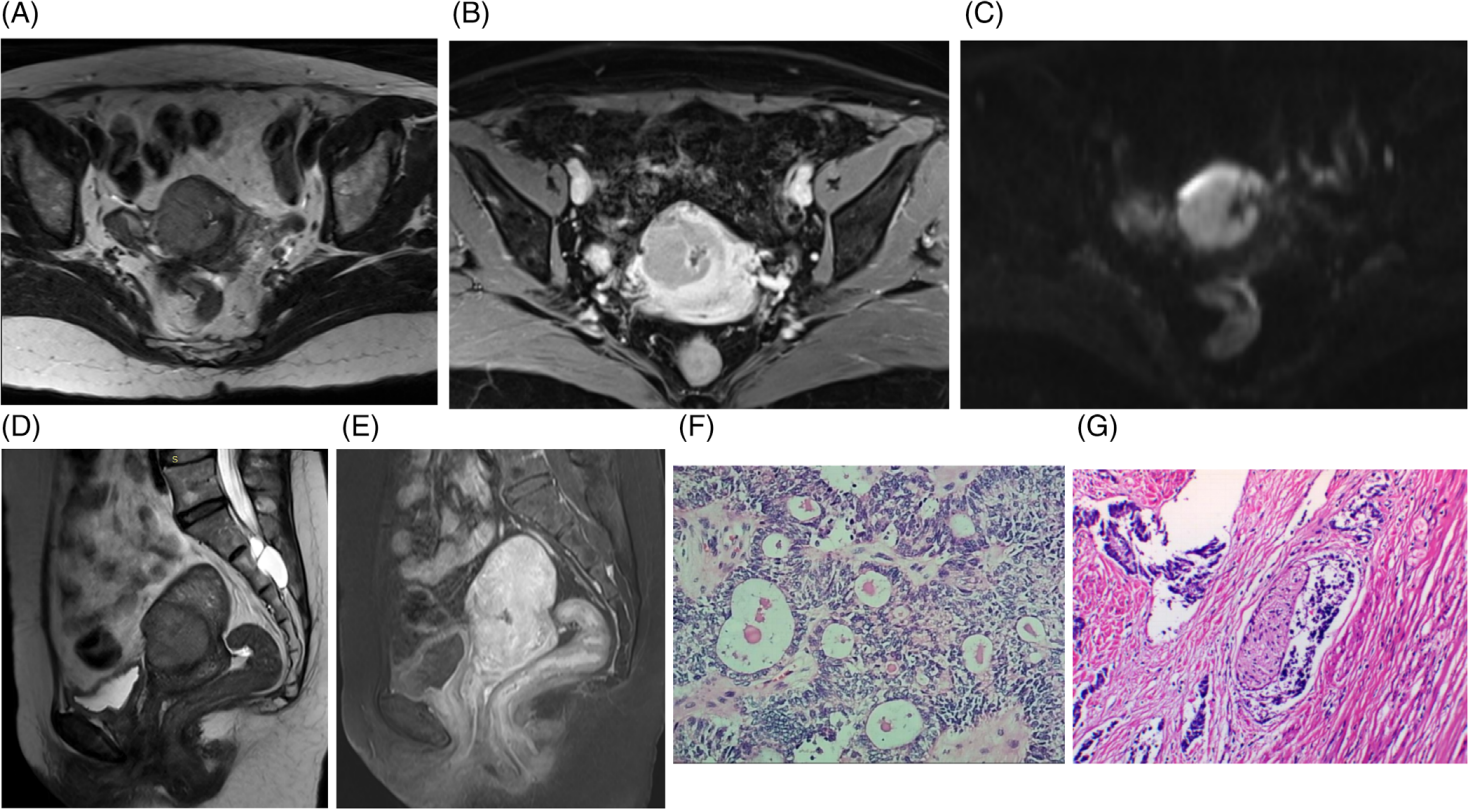
MRI and pathological images of reported MC case. (A)Transverse T2WI, (B) transverse T1WI enhancement, (C) transverse DWI, (D) sagittal T2WI, (E) sagittal T1WI enhancement, (F) low‐power HE‐stained microscopic image, and (G) high‐power HE‐stained microscopic image.

A 53‐year‐old woman who has been postmenopausal for 1 year presented with no clinical symptoms. During her annual routine check‐up, elevated levels of CA‐125 (44.99 U/mL) and HE‐4 (173.3 pmol/L) were detected. Gynecological ultrasound revealed heterogeneous echogenicity within the uterine cavity, with an irregularly shaped mass measuring approximately 55 × 40 × 30 mm and displaying low echogenicity. No significant abnormalities were observed in the bilateral adnexa. Hysteroscopy indicated adhesions in the upper segment of the cervical canal, abnormal uterine cavity morphology, thickened endometrium, and vascular engorgement. Pathological examination following uterine segmental curettage revealed small round cell malignancy in the endocervical and endometrial epithelium. Pelvic magnetic resonance imaging (MRI) confirmed a uterine mass (31 × 36 × 44 mm) protruding into the myometrium at the right anterior margin of the uterus, raising suspicion of endometrial carcinoma(Figure 2A–E). Multiple small lymph nodes were identified in the pelvic region and inguinal area, along with multiple nabothian cysts. Chest CT scan showed minimal fibrous lesions in the right middle lung and a few bullae in the right middle and lower lung. The patient's obstetric history included one vaginal delivery and one miscarriage, and she had no previous history of tumors or genetic diseases.

The patient underwent a laparoscopic total hysterectomy with bilateral salpingo‐oophorectomy and pelvic lymph node dissection. During surgery, low‐grade carcinoma was discovered in the superficial myometrial layer of the uterus via frozen section analysis. The final pathology report indicated a primary MC originating from the cervix primarily located at the junction of the lower segment of the uterus and cervix. The tumor did not breach the serosal surface, but extensive infiltration was observed with glandular differentiation. Immunohistochemistry studies revealed positive results for Syn, cgA, CD56, and Vimentin, while negative for D2‐40, Calretinin, Inhibin‐α, CD99, AR, P16, CEA, MC, CK5/6, W‐1. Additionally, the Ki‐67 index was 20%–30%. Elastic fiber staining was negative. Typical microscopic pathological photos are shown in Figure 2F,G. No significant tumor was found in the bilateral adnexa. The lymph nodes on the left side showed no evidence of metastasis (0/7), as well as the lymph nodes on the right side (0/3). Microscopic examination of the peritoneal deposit in the rectovesical pouch revealed fibrous tissue with crystal deposits and foreign body giant cell reaction, without evidence of tumor. The patient was staged as FIGO IB2. She received 6 cycles of neoadjuvant chemotherapy with docetaxel and carboplatin. Regular follow‐up for 4 years has shown no signs of tumor recurrence.

### 4.2 Patient characteristics

**FIGURE 3:**
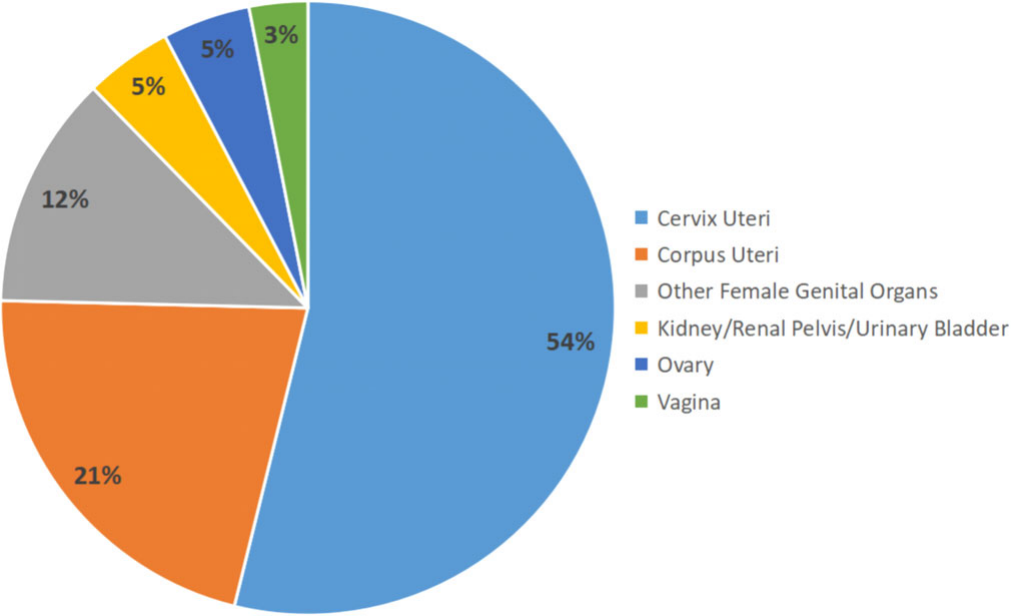
The distribution of primary sites of MC.

A total of 65 MC patients, who were initially diagnosed between 2005 and 2019 were finally enrolled in the present study. Among those MC patients, 15 (23.08%) patients died from MC, and 6 (9.23%) patients died from other causes, with the average survival time at 84.22 ± 50.66 months, up to date. Among those patients, the most common primary site was cervix uteri (53.85%), followed by corpus uteri (21.54%), other female genital organs (12.31%), ovary (4.62%), kidney/renal pelvis/urinary bladder(4.62%), and vagina(3.08%). 37 (56.92%) patients were diagnosed at stage FIGO I, while 17 (26.15%) at FIGO II, 7 (10.77%) at FIGO III, and 3 (4.62%) at FIGO IV. While being staged according to the SEER stage standard, 33 (50.77%) patients were staged localized, 24 (36.92%) were regional, and 8 (12.31%) were distant. The distribution of the primary site of MC is shown in Figure 3.

The detailed information on those MC patients as well as the difference between the development and the validation cohorts were summarized in Table 1. Tables S1, S2, and S3 showed detailed information on those patients who underwent surgery, radiotherapy, and chemotherapy.

**TABLE 1:** Patient characteristics and clinicopathological variables.

Variables	Total	Cohort		*p* Value
		Development	Validation	
*N*	65	47	18	
*Survival months*	84.2 ± 50.66	82.2 ± 50.1	89.4 ± 53.1	.671
*Age*		54.5 ± 13.3	55.7 ± 11.4	.907
*Age group*				.289
≤40	7 (10.77%)	6 (12.8%)	1 (5.6%)	
41–49	11 (16.92%)	6 (12.8%)	5 (27.8%)	
50–59	24 (36.92%)	20 (42.6%)	4 (22.2%)	
60–69	15 (23.08%)	9 (19.1%)	6 (33.3%)	
70+	8 (12.31%)	6 (12.8%)	2 (11.1%)	
*Race*				.808
White	48 (73.85%)	34 (72.3%)	14 (77.8%)	
Black	6 (9.23%)	4 (8.5%)	2 (11.1%)	
Other	11 (16.92%)	9 (19.1%)	2 (11.1%)	
*Primary site*				.387
Cervix uteri	35 (53.85%)	24 (51.1%)	11 (61.1%)	
Corpus uteri	14 (21.54%)	12 (25.5%)	2 (11.1%)	
Other female genital organs	8 (12.31%)	7 (14.9%)	1 (5.6%)	
Ovary	3 (4.62%)	1 (2.1%)	2 (11.1%)	
Kidney/renal pelvis/ urinary bladder	3 (4.62%)	2 (4.3%)	1 (5.6%)	
Vagina	2 (3.08%)	1 (2.1%)	1 (5.6%)	
*Tumor differentiated grade*				.656
Unknown	26 (40.00%)	17 (36.2%)	9 (50.0%)	
Well differentiated; Grade I	14 (21.54%)	12 (25.5%)	2 (11.1%)	
Moderately differentiated; Grade II	16 (24.62%)	12 (25.5%)	4 (22.2%)	
Poorly differentiated; Grade III	7 (10.77%)	5 (10.6%)	2 (11.1%)	
Undifferentiated; anaplastic; Grade IV	2 (3.08%)	1 (2.1%)	1 (5.6%)	
*SEER stage*				.587
Localized	33 (50.77%)	24 (51.1%)	9 (50.0%)	
Regional	24 (36.92%)	16 (34.0%)	8 (44.4%)	
Distant	8 (12.31%)	7 (14.9%)	1 (5.6%)	
*Duration from diagnosis to treatment*				.220
Less than 1 month	43 (66.15%)	29 (61.7%)	14 (77.8%)	
More than 1 month	22 (33.85%)	18 (38.3%)	4 (22.2%)	
*Surgery*				
Surgery performed	61 (93.85%)	45 (95.7%)	16 (88.9%)	.305
Not recommended	4 (6.15%)	2 (4.3%)	2 (11.1%)	
*Chemotherapy*				.220
No	43 (66.15%)	29 (61.7%)	14 (77.8%)	
Yes	22 (33.85%)	18 (38.3%)	4 (22.2%)	
*Radiotherapy*				.711
No radiotherapy	41 (63.08%)	29 (61.7%)	12 (66.7%)	
Radiotherapy prior or after surgery	24 (36.92%)	18 (38.3%)	6 (33.3%)	
*Lymph nodes resection*				.209
No	28 (43.08%)	18 (38.3%)	10 (55.6%)	
Yes	37 (56.92%)	29 (61.7%)	8 (44.4%)	
*Regional LN examined*				.402
None	25 (38.46%)	16 (34.0%)	9 (50.0%)	
≤10	11 (16.92%)	10 (21.3%)	1 (5.6%)	
11–20	24 (36.92%)	17 (36.2%)	7 (38.9%)	
≥21	5 (7.69%)	4 (8.5%)	1 (5.6%)	
*Regional LN positive*				.576
0	35 (53.85%)	27 (57.4%)	8 (44.4%)	
More than 1	5 (7.69%)	4 (8.5%)	1 (5.6%)	
No LN examined	25 (38.46%)	16 (34.0%)	9 (50.0%)	
*Bone metastasis*				.533
No	64 (98.46%)	46 (97.9%)	18 (100.0%)	
Yes	1 (1.54%)	1 (2.1%)	0 (0.0%)	
*Lung metastasis*				.480
No	63 (96.92%)	46 (97.9%)	17 (94.4%)	
Yes	2 (3.08%)	1 (2.1%)	1 (5.6%)	
*Tumor size*				.416
≤5 cm	36 (55.38%)	27 (57.4%)	9 (50.0%)	
>5 cm	11 (16.92%)	9 (19.1%)	2 (11.1%)	
Unknown	18 (27.69%)	11 (23.4%)	7 (38.9%)	
*FIGO stage*				.092
FIGO I	37 (56.92%)	27 (57.4%)	10 (55.6%)	
FIGO II	17 (26.15%)	10 (21.3%)	7 (38.9%)	
FIGO III	7 (10.77%)	7 (14.9%)	0 (0.0%)	
FIGO IV	3 (4.62%)	3 (6.4%)	0 (0.0%)	
Unknown	1 (1.54%)	0 (0.0%)	1 (5.6%)	
*Cancer‐specific death*				.919
Alive	50 (76.92%)	36 (76.6%)	14 (77.8%)	
Dead	15 (23.08%)	11 (23.4%)	4 (22.2%)	
*Cancer‐competitive death*				.799
Alive	44 (67.69%)	31 (66.0%)	13 (72.2%)	
Die for cancer	15 (23.08%)	11 (23.4%)	4 (22.2%)	
Die for other cause	6 (9.23%)	5 (10.6%)	1 (5.6%)	

* Statistically significant (*p* < .05).

### 4.3 Kaplan–Meier analysis

**FIGURE 4:**
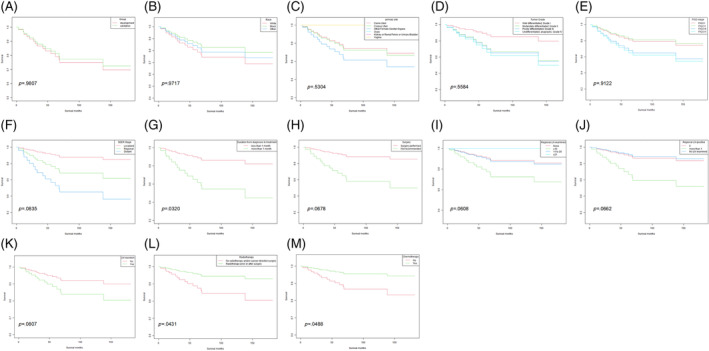
The Kaplan–Meier analyses of different subgroups of MC patients. (A) Development and validation cohort, (B) race, (C) primary site, (D) tumor differentiated grade, (E) FIGO stage, (F) SEER stage, (G) duration from diagnosis to begin treatment, (H) surgery, (I) regional lymph nodes examined, (J) regional lymph nodes positive, (K) regional lymph nodes resection, (L) radiotherapy, and (M) chemotherapy.

As is shown in Figure 4A, there is no significant difference in the OS in those patients between the development and the validation cohorts (*p* = .9807). Different groups of race, primary site, tumor differentiated grade, and the FIGO stage also showed no different OS in the Kaplan–Meier analysis (Figure 4B–E). However, a significant difference was found in patients' survival time when MC patients were staged by the SEER stage standards as localized, regional, or distant, which indicates that the SEER stage standards might be a better staging system in the MC patients to distinguish their prognosis (Figure 4F, *p* = .0835).

Additional Kaplan–Meier analyses were applied to the effect of different treatments on the survival time in those patients. We first analyzed the influence of the waiting time from diagnosis to begin treatment. As is shown in Figure 4G, patients benefited from less than 1 month's waiting to begin their treatment (*p* = .0320). Figure 4H showed that those who accepted surgery had a better survival time (*p* = .0678). We also compared the influence of different solutions of regional lymph nodes and found that MC patients benefited from regional lymph node examination (Figure 4I, *p* = .0608). Moreover, patients who found more than 1 regional lymph node positive (Figure 4J, *p* = .0662), or received regional lymph node resection (Figure 4K, *p* = .0807) had a poorer survival time. Radiotherapy (Figure 4L, *p* = .0431), as well as chemotherapy (Figure 4M, *p* = .0488), showed a significant influence on the survival time in the MC patients.

### 4.4 Univariate and multivariate Cox regression analysis with cancer‐specific death

Univariate and multivariate analyses were applied to estimate the effect of those variables on cancer‐specific death. As is shown in Table 2, the SEER stage, more than 1‐month waiting from diagnosis to begin treatment, surgery performed, ≤10 lymph nodes examined, regional lymph positive, lung metastasis, and tumor size larger than 5 cm showed significant influence on cancer‐specific death through univariate analysis. As for the multivariate Cox regression analysis, age at the year of diagnosis, race, primary site, tumor differentiated grade, the SEER stage, more than 1‐month waiting from diagnosis to begin treatment, surgery, chemotherapy, radiotherapy, lymph node resection/examination/positive, lung metastasis, the FIGO stage shown significant influence to the cancer‐specific death in MC patients.

**TABLE 2:** Univariate and multivariate analysis of specific cancer death.

Variables	Univariate analysis		Multivariate analysis	
	HR (95% CI)	*p* Value	HR (95% CI)	*p* Value
*Age*	1.00 (0.96, 1.04)	.991	0.39 (0.37, 0.41)	<.001*
*Age group*				
≤40	1.0		1.0	
41–49	0.40 (0.07, 2.40)	.317	165.13 (38.03, 717.02)	<.001*
50–59	0.39 (0.09, 1.74)	.216	13665.78 (3378.35, 55279.45)	<.001*
60–69	0.78 (0.17, 3.50)	.742	inf. (inf., inf.)	<.001*
70+	0.67 (0.11, 4.05)	.665	inf. (inf., inf.)	<.001*
*Race*				
White	1.0		1.0	
Black	0.66 (0.09, 5.06)	.687	0.01 (0.00, 0.09)	<.001*
Other	0.83 (0.18, 3.70)	.802	0.32 (0.07, 1.48)	.145
*Primary site*				
Cervix uteri	1.0		1.0	
Corpus uteri	1.13 (0.30, 4.22)	.858	97.56 (22.22, 428.40)	<.001*
Other female genital organs	1.64 (0.44, 6.06)	.459	7.13 (1.46, 34.69)	.015*
Ovary	0.00 (0.00, Inf)	.999	0.00 (0.00, 2.73)	.086
Kidney/renal pelvis/urinary bladder	0.00 (0.00, Inf)	.994	9.13 (0.00, 562767.06)	.694
Vagina	0.00 (0.00, Inf)	.999	0.00 (0.00, 0.27)	.026*
*Tumor differentiated grade*				
Unknown	1.0		1.0	
Well differentiated; Grade I	0.88 (0.16, 4.84)	.887	302.39 (40.05, 2283.11)	<.001*
Moderately differentiated; Grade II	2.22 (0.63, 7.88)	.217	30.08 (8.78, 103.07)	<.001*
Poorly differentiated; Grade III	2.50 (0.45,13.83)	.292	21380.23 (2348.95, 194604.04)	<.001*
Undifferentiated; anaplastic; Grade IV	2.94 (0.33,26.41)	.336	34.92 (3.24, 376.06)	.003*
*SEER stage*				
Localized	1.0		1.0	
Regional	2.75 (0.81, 9.42)	.106	475.42 (137.72, 1641.16)	<.001*
Distant	6.06 (1.50,24.42)	.011*	1028.86 (249.44, 4243.65)	<.001*
*Duration from diagnosis to treatment*				
Less than 1 month	1.0		1.0	
More than 1 month	3.89 (1.37,11.06)	.011*	1917.74 (577.63, 6366.95)	<.001*
*Surgery*				
Surgery performed	1.0		1.0	
Not recommended	7.85 (1.72,35.81)	.008*	45050.88 (4515.93, 449427.54)	<.001*
*Chemotherapy*				
No	1.0		1.0	
Yes	0.75 (0.24, 2.37)	.627	15.18 (3.63, 63.46)	.002*
*Radiotherapy*				
No radiotherapy	1.0		1.0	
Radiotherapy prior or after surgery	0.35 (0.10, 1.22)	.100	0.00 (0.00, 0.00)	<.001*
*Lymph nodes resection*				
No	1.0		1.0	
Yes	2.05 (0.65, 6.47)	.219	0.00 (0.00, 0.02)	<.001*
*Regional LN examined*				
None	1.0		1.0	
≤10	3.45 (0.97,12.26)	.056*	11249.68 (3248.04, 38963.62)	<.001*
11–20	1.19 (0.32, 4.45)	.796	0.72 (0.22, 2.39)	.589
≥21	0.00 (0.00, Inf)	.998	0.00 (0.00, 0.00)	<.001*
*Regional LN positive*				
0	1.0		1.0	
More than 1	6.07 (1.73,21.34)	.005*	9.62 (1.94, 47.60)	.006*
No LN examined	0.91 (0.27, 3.11)	.881	1.00 (0.23, 4.33)	1.000
*Bone metastasis*				
No	1.0		1.0	
Yes	0.00 (0.00, Inf)	.998	252.88 (0.00, inf.)	.672
*Lung metastasis*				
No	1.0		1.0	
Yes	inf. (0.00, Inf)	.018*	0.05 (0.01, 0.26)	.001*
*Tumor size*				
≤5 cm	1.0		1.0	
>5 cm	3.29 (1.07, 10.13)	.038*	0.33 (0.08, 1.41)	.135
Unknown	0.21 (0.03, 1.68)	.142	0.47 (0.06, 3.37)	.450
*FIGO Stage*				
FIGO I	1.0		1.0	
FIGO II	0.89 (0.24, 3.36)	.864	0.03 (0.01, 0.13)	<.001*
FIGO III	1.84 (0.39, 8.68)	.443	0.00 (0.00, 0.00)	<.001*
FIGO IV	2.04 (0.25,16.40)	.503	1.43 (0.07, 30.41)	.817
Unknown	4.56 (0.56,36.97)	.155	0.00 (0.00, 0.00)	<.001*
*Cohort*				
Development	1.0		1.0	
Validation	0.89 (0.28, 2.81)	.845	0.39 (0.10, 1.49)	.168

* Statistically significant (*p* < .05).

### 4.5 Nomogram construction and validation

**FIGURE 5:**
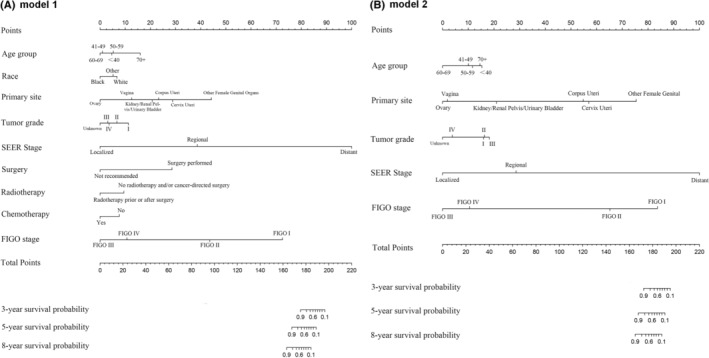
Predicting nomogram. (A) Nomogram model 1 based on the variables selected by the univariate and multivariate cox regression analyses. (B) Nomogram model 2 based on age, primary site, tumor differentiated grade, SEER and FIGO stage.

**FIGURE 6:**
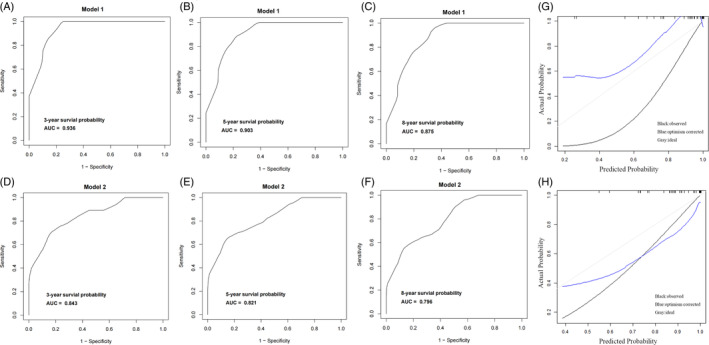
ROC and calibration curves. (A–C) ROC curves of model 1 to predict 3‐to‐8‐year survival probability of MC patients. (D–F) ROC curves of model 2 to predict 3‐to‐8‐year survival probability of MC patients. (G) Calibration curve of model 1. (H) Calibration curve of model 2.

**FIGURE 7:**
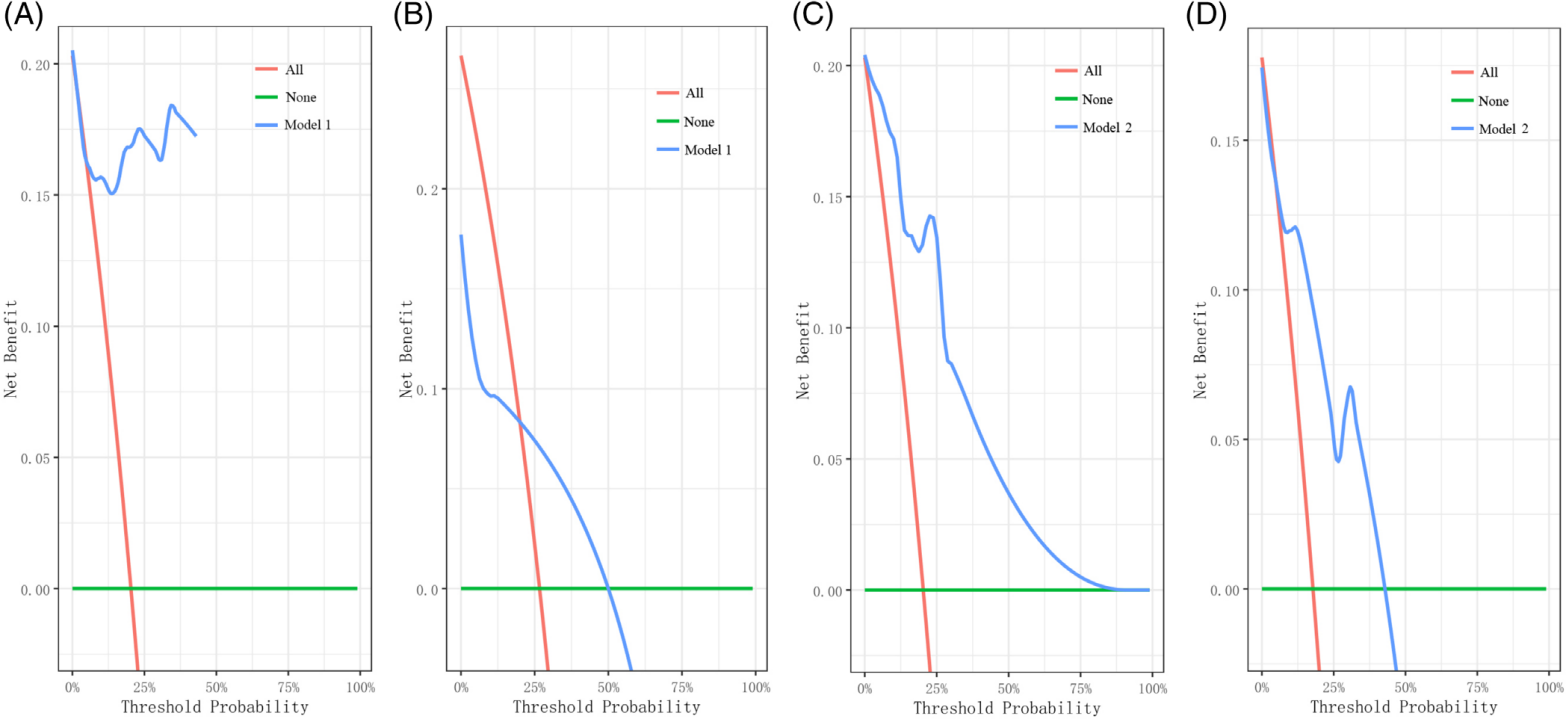
DCA curves. (A) DCA curve of model 1 in development cohort. (B) DCA curve of model 1 in validation cohort. (C) DCA curve of model 2 in development cohort. (D) DCA curve of model 2 in validation cohort.

The first nomogram model 1 was established based on those significant variables selected by the multivariate Cox regression analysis, which were age, race, primary site, tumor differentiated grade, the SEER stage, surgery, radiotherapy, chemotherapy, and the FIGO stage to predict the 3‐to‐8‐year survival probability of MC patients (Figure 5A). Meanwhile, a simplified nomogram model 2 was established based on age, the primary site of the tumor, tumor differentiated grade, the SEER stage, and the FIGO stage for patients' estimation before treatment (Figure 5B). The C‐index of model 1 was 0.921 in the development cohort and 0.720 in the validation cohort, while model 2 had a C‐index of 0.848 in the development cohort and 0.640 in the validation cohort. Model 1 achieved a good predictive value in the ROC analyses to predict a 3‐year (Figure 6A, AUC = 0.936), 5‐year (Figure 6B, AUC = 0.903), and 8‐year (Figure 6C, AUC = 0.875). Moreover, model 2 also achieved a good predictive value in the ROC analyses to predict a 3‐year (Figure 6D, AUC = 0.843), 5‐year (Figure 6E, AUC = 0.821), and 8‐year (Figure 6F, AUC = 0.796) survival probability. The calibration curves showed both model 1 (Figure 6G) and model 2 (Figure 6H) had good agreement between predicted probability and the observed outcome. The DCA analyses also showed that model 1 (Figure 7A,B) and model 2 (Figure 7C,D) had good discrimination both in the development and validation cohort.

**FIGURE 8:**
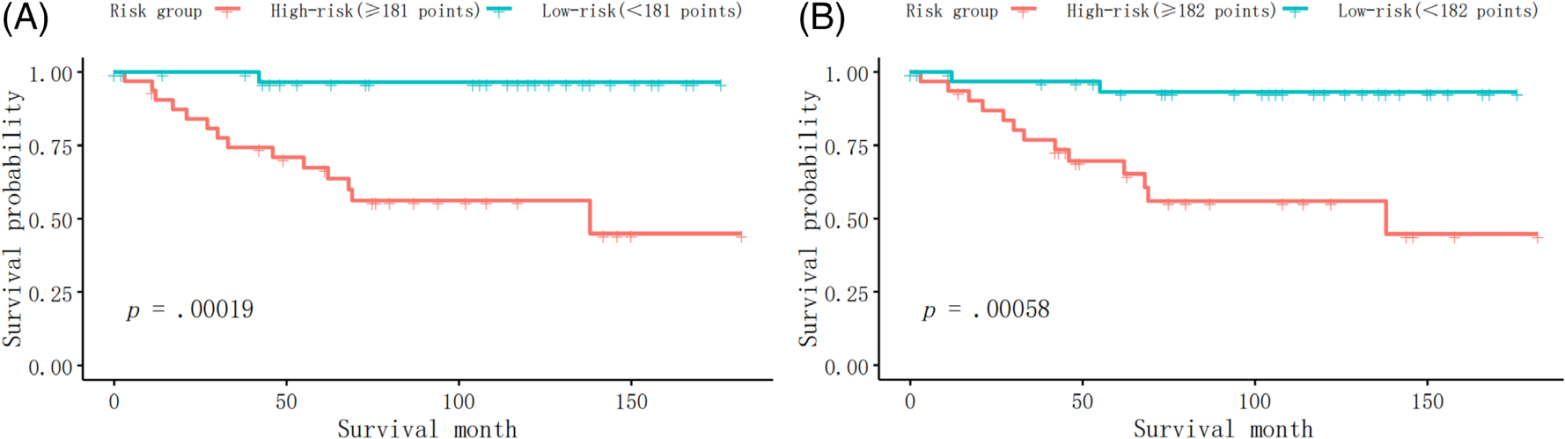
Survival curves of MC patients in different risk groups. (A) The survival curve of high‐risk and low‐risk MC patients selected by the nomogram model 1. (B) The survival curve of high‐risk and low‐risk MC patients selected by the nomogram model 2.

Furthermore, we identified the patients into low‐risk and high‐risk groups at the dichotomous quantiles of total points of the nomogram total points in the whole cohort, which cut‐off points were 181 in model 1 and 182 in model 2, respectively, and compared the difference of the survival time between them. Figure 8A showed that there was a significant difference in survival time between the high‐risk and low‐risk patients (*p* = .00019), based on the nomogram model 1. Meanwhile, Figure 8B also showed the significant survival time difference between the high‐risk and low‐risk patients (*p* = .00058), based on the nomogram model 2.

Integrated discrimination improvement (IDI) and net reclassification improvement (NRI) were enrolled to compare the predicting and discrimination capability between model 1 and model 2. As is shown in Table 3, there was no significant difference between model 1 and model 2 in predicting the 3‐year survival probability (IDI = −0.261, *p* = .897; NRI = −0.479, *p* = .226), the 5‐year survival probability (IDI = −0.285, *p* = .837; NRI = −0.634, *p* = .286), the 8‐year survival probability (IDI = −0.255, *p* = .844; NRI = −0.524, *p* = .166). The detailed points of these two nomograms are shown in Figures S1 and S2.

**TABLE 3:** Comparison between model 1 and model 2.

Predict probability	IDI	*p* Value	NRI	*p* Value
3‐year survival probability	−0.261	.897	−0.479	.226
5‐year survival probability	−0.285	.837	−0.634	.286
8‐year survival probability	−0.255	.844	−0.524	.166

* Statistically significant (*p* < .05).

## 5 DISCUSSION

MC is a very rare disease with causes and optimal management strategies that are incompletely characterized and understood. Because of the limitation of cases, large randomized prospective research of MC is impossible to carry out, leaving a lack of clinical guidelines and expert consensus for clinicians and patients in practice. In this study, we derived the largest cohort of MC patients so far, as well as carried out additional analyses to present the characteristics of MC patients. To clarify, the significance level of the present study was set as *p* < .1 in the Kaplan–Meier analyses, and *p* < .05 in univariate and multivariate Cox regression analyses as mentioned above.

As is shown in Table 1, the average survival time of MC patients was 84.2 ± 50.66 months, with more than 50% of patients diagnosed at stage FIGO I or localized at the SEER staging system. Dinh et al. summarized 67 reported MC cases, approximately 70% of cases are localized to the cervix (FIGO IB).^10^ The primary sites of MC were summarized in Figure 3, which indicated that the staging and the treatment of male MC patients should mainly follow the guidelines of gynecologic tumors. Federico Ferrari et al. reported an MC of the vagina and conducted a literature review recently.^23^ Surgery was the primary treatment in most cases of MC of the vagina, which showed satisfied prognosis with no recurrence case reported. Our study also included two cases of MC of the vagina extracted from the SEER database mentioned above. Figure 4C showed that the MC of the vagina seems to have a better prognosis than other localizations of MC, which might be contributed by the certain anatomical structure and lymphatic drainage. Those MC patients with well‐differentiated tumor grades might had a better OS than others (Figure 4D). Further research is needed to explore the biological behavior and lymphatic drainage patterns of MC.

Our Kaplan–Meier analysis showed different FIGO stages did not distinguish different survival outcomes in MC patients (Figure 4E, *p* = .9122), though patients staged as FIGO I shared a similar OS with those staged as FIGO II, as well as the survival outcomes for patients with FIGO III stage were comparable to those with FIGO IV stage. Meanwhile, different patients of different SEER stages did have significantly different OS (Figure 4F, *p* = .0835), which indicated the SEER stage might be a better staging system in MC patients.

As for the treatment, patients benefited from a shorter waiting time to begin their treatment (Figure 4G, *p* = .0320), which has been also confirmed by the multivariate Cox regression analysis (Table 2, *p* < .001). Thus, we recommend a shorter duration from diagnosis to begin treatment in MC patients.

More than 90% of MC patients underwent surgery, indicating surgery is the main treatment. The details of patients who underwent surgery or not were summarized and compared in Table S1. The median OS of patients receiving surgery was 54.7 ± 13.0 months while those who did not receive surgery were 57.5 ± 5.9 months, no significant difference was observed between these two groups (*p* = .672). Nevertheless, after adjusting age and the SEER stage as mentioned above, Figure 4H showed those patients who underwent surgery had a better OS. The decision to perform surgery on a patient is dependent on multiple factors, including staging, surgical tolerance, and individualized considerations. The impact of specific surgical interventions on patient prognosis needs to be analyzed while considering baseline characteristics such as age and clinical staging. We assumed that surgical treatment may be a valuable option for MC patients.

In summarizing 39 cases of MC, Dierickx found that treatment varied based on disease stage, with options including hysterectomy (HRT) with or without bilateral salpingo‐oophorectomy (BSO), pelvic lymphadenectomy (LA) and (neo‐) adjuvant chemo‐ or radiotherapy.^11^ However, the biological behavior of this unusual tumor remains unclear and until there is sufficient data to recommend a particular course of therapy it seems reasonable to manage patients with MC of the cervix according to current guidelines for cervical adenocarcinoma of similar stage. Additional Kaplan–Meier analysis showed that patients benefited from the examination of regional lymph nodes (Figure 4I, *p* = .0608). Regional lymph nodes positive (Figure 4J, *p* = .0662), as well as regional lymph nodes resection (Figure 4K, *p* = .0807), was related to a poorer OS. Moreover, the multivariate Cox regression analysis also showed that patients benefited from regional lymph node resection (HR = 0, *p* < .001), and more than 21 lymph nodes were examined (HR = 0, *p* < .001). Catarina's report described a 60‐year‐old woman with FIGO IB1 stage endometrial MC who underwent a radical hysterectomy and had 28 lymph nodes that tested negative for the presence of tumors. The patient also received adjuvant radiotherapy after surgery. However, as the lack of guidelines, the optimal strategy of lymph node resection in MC remains unknown.^24^ Based on our findings, a thorough examination of regional lymph nodes and extensive resection during surgery in MC patients may confer significant benefits.

Additional Kaplan–Meier analyses showed that radiotherapy performed prior or/and after surgery showed a tendency to benefit patients' survival (Figure 4L, *p* = .0431). However, the optimal radiotherapy paradigm of MC patients remains unknown. Dinh et al reported a case of locally advanced, node‐positive cervical MC in a 55‐year‐old woman who received both external radiotherapy and brachytherapy techniques but failed to summarize the optimal radiotherapy strategy.^10^ Since there is limited research that has specifically focused on the effect of radiotherapy in MC, it remains unknown whether MC is a radio‐sensitive tumor or not. The beneficial effect of radiotherapy shown in our study indicated that MC is a kind of radio‐sensitive rather than a radio‐resistant tumor. Iris Mueller reported a case of vaginal MC in a 54‐year‐old woman, who underwent R0 resection. Adjuvant radiochemotherapy was then administered, which involved external radiation and brachytherapy along with 5 cycles of cisplatin (40 mg/m^2^) for radiosensitization. After 4 years of continuous oncologic follow‐up, the patient remains alive and there are no signs of disease.^15^ Samar Shoeir reported that a 63‐year‐old woman diagnosed with MC completed a course of prophylactic brachytherapy after resection to prevent possible recurrence of the disease.^16^ It is worth noting that this case report represents only one patient's experience and may not be generalizable to all cases of MC. Further research is needed to determine the effectiveness of radiotherapy in treating this rare type of cancer, as well as explore the best radiotherapy strategy in MC patients, including the optimal biologically effective dose (BED), treatment volume, as well as the number of fractions.

Patients who accepted chemotherapy had a poorer OS in the multivariate Cox regression analysis (HR = 15.18, *p* = .002), which might be attributed to the high portion of advanced stages (Table S3). Nevertheless, our Kaplan–Meier analysis demonstrated a survival benefit in patients who received chemotherapy after considering age and clinical stage as adjusted variables (Figure 4M). As chemotherapy was thought to be the basic therapy for many carcinomas, additional research is warranted to elucidate the impact of chemotherapy on MC patients, as well as to explore any potential synergistic effects when combined with radiotherapy.

Nomograms have been widely used for the prediction of certain outcomes clinically for their convenience and reliability. Due to the rarity of MC, no guideline had been published so that nomogram could play an important role in the management of MC patients. Here in the present study, to our knowledge, we for the first time to construct predictive models to predict the survival probability of MC patients, including both of the treatment information and the clinicopathological parameters as variables. We first constructed a nomogram model 1 based on those variables showing significant influence the cancer‐specific death to predict the 3‐to‐8‐year survival probability (Figure 5A). Additionally, we constructed a simplified nomogram model 2 based on only five variables which clinicians would attach importance, to predict the same 3‐to‐8‐year survival probability in MC patients (Figure 5B). Both the two nomograms got satisfied scores in the C‐index, ROC curves, DCA curves, and calibration plots, which could be recommended in predicting the survival probability and sorting those high‐risk patients in MC patients.

As MC is rare in male and only four male patients had been recorded in the SEER database with incomplete information as mentioned above, male patients were excluded in the present study to avoid bias when constructing predicting models. Currently, there is a paucity of research regarding male MC patients, with only a few published studies available. Pranjal Agrawal et al. reported a case of a 63‐year‐old man with MC of the bladder, who underwent complete resection of the tumor using bipolar electrocautery and fulguration of the base.^19^ Another male MC case was described by Konnak in 1980.^25^ No predictive model has been constructed for male MC patients so far. Further research is warranted to better understand the risk factors associated with male MC patients and to consolidate knowledge in this area.

Our study has several limitations. First, we excluded 462 cases due to incomplete information or repeating records from the SEER database, which reduced the scale of our research. Second, being a retrospective study, we faced some data limitations such as missing information about surgery extent, chemotherapy protocol, radiotherapy technicalities, and patient treatment response, which might lead to bias. Thirdly, we lacked detailed information on pathology reports such as CEA, HR, Ki‐67 expression levels, and tumor genetics, which could have influenced tumor behavior. More prospective studies are needed to address these limitations. Nevertheless, because of the rarity of MC, it is impossible to include more patients in the present study, which to our knowledge is the largest study in MC patients.

## 6 CONCLUSION

Our study reviewed, summarized, and analyzed the characteristics of MC patients by including 65 MC patients to construct the largest MC patients' cohort up to date. Sufficient regional lymph nodes examined, as well as applying radiotherapy prior to or after in high‐risk patients are recommended in MC patients. This study also for the first time constructed an external validation nomogram to predict the survival probability of MC patients, which had good predicting and discriminating capability and was helpful in patients' individual risk estimation, management, counseling, and follow‐up.

## AUTHOR CONTRIBUTIONS

**Zhuoran Li:** Data curation (equal); methodology (equal); project administration (equal). **Dongyu Liu:** Conceptualization (equal); supervision (equal). **Wenlong Wei:** Visualization (equal); writing – original draft (equal). **Zhisheng Huang:** Project administration (equal); supervision (equal); validation (equal). **Yuzhen Mo:** Project administration (lead); writing – original draft (lead); writing – review and editing (lead). **Haowei Huang:** Conceptualization (lead); data curation (lead); formal analysis (lead); writing – review and editing (lead).

## FUNDING INFORMATION

This study was funded by the Guangzhou Health Science and Technology project (grant number 20221A010014, 20241A011022), the Guangzhou Science and Technology Bureau Program (grant number 202201010806), the Project of Guangdong Medical Science and Technology Research (grant number A2023469), the Huadu District Science and Technology Plan Project (grant number 21‐HDWS‐066).

## CONFLICT OF INTEREST STATEMENT

The authors report no conflict of interest.

## Supporting information


**Figure S1.** Detailed points of the variables in nomogram model 1

**Figure S2.** Detailed points of the variables in nomogram model 2

**Table S1.** Patient characteristics and clinicopathological variables with or without surgery performed

**Table S2.** Patient characteristics and clinicopathological variables with or without radiotherapy performed

**Table S3.** Patient characteristics and clinicopathological variables with or without chemotherapy performed

## Data Availability

The data of the present study is available on request from the corresponding authors.
