# Supplementary material for: Identification of characteristics and construction of nomogram to predict the survival probability of mesonephric carcinoma patients: A population‐based analysis and a case report
Source: Cancer Rep (Hoboken). 2023 Nov 29;7(1):e1940. doi: 10.1002/cnr2.1940 (PMC10809193; doi:10.1002/cnr2.1940)
Supplement: Supplementary file 1 — Figure S1. Detailed points of the variables in nomogram model 1 [file CNR2-7-e1940-s002.doc]

**Supplementary Figure 1.**

**Detailed points of the variables in nomogram model 1**

| **Age** | points |
| --- | --- |
| ＜40 | 5 |
| 41-49 | 1 |
| 50-59 | 5 |
| 60-69 | 0 |
| 70+ | 16 |
|  |  |
| **Race** | points |
| White | 7 |
| Black | 0 |
| Other | 5 |
|  |  |
| **Primary site** | points |
| Cervix Uteri | 29 |
| Corpus Uteri | 23 |
| Other Female Genital Organs | 44 |
| Ovary | 0 |
| Kidney or Renal Pelvis or Urinary Bladder | 21 |
| Vagina | 13 |
|  |  |
| **Tumor differentiated Grade** | points |
| Unknown | 0 |
| Well differentiated; Grade I | 11 |
| Moderately differentiated; Grade II | 7 |
| Poorly differentiated; Grade III | 3 |
| Undifferentiated; anaplastic; Grade IV | 4 |
|  |  |
| **SEER Stage** | points |
| Localized | 0 |
| Regional | 39 |
| Distant | 100 |
|  |  |
| **Surgery** | points |
| Surgery performed | 29 |
| Not recommended | 0 |
|  |  |
| **Radiotherapy** | points |
| No radiotherapy | 9 |
| Radiotherapy prior or after surgery | 0 |
|  |  |
| **Chemotherapy** | points |
| No | 8 |
| Yes | 0 |
|  |  |
| **FIGO Stage** | points |
| FIGO I | 72 |
| FIGO II | 44 |
| FIGO III | 0 |
| FIGO IV | 11 |
|  |  |
|  |  |
| **Total Points** | **Probability for 3-year survival** |
| 197 | 0.1 |
| 194 | 0.2 |
| 192 | 0.3 |
| 190 | 0.4 |
| 188 | 0.5 |
| 186 | 0.6 |
| 184 | 0.7 |
| 180 | 0.8 |
| 175 | 0.9 |
|  |  |
| **Total Points** | **Probability for 5-year survival** |
| 189 | 0.1 |
| 187 | 0.2 |
| 185 | 0.3 |
| 183 | 0.4 |
| 181 | 0.5 |
| 179 | 0.6 |
| 176 | 0.7 |
| 173 | 0.8 |
| 168 | 0.9 |
|  |  |
| **Total Points** | **Probability for 8-year survival** |
| 184 | 0.1 |
| 182 | 0.2 |
| 180 | 0.3 |
| 178 | 0.4 |
| 176 | 0.5 |
| 174 | 0.6 |
| 172 | 0.7 |
| 168 | 0.8 |
| 163 | 0.9 |
