# Supplementary material for: Identification of characteristics and construction of nomogram to predict the survival probability of mesonephric carcinoma patients: A population‐based analysis and a case report
Source: Cancer Rep (Hoboken). 2023 Nov 29;7(1):e1940. doi: 10.1002/cnr2.1940 (PMC10809193; doi:10.1002/cnr2.1940)
Supplement: Supplementary file 2 — Figure S2. Detailed points of the variables in nomogram model 2 [file CNR2-7-e1940-s001.doc]

**Supplementary Figure 2.**

**Detailed points of the variables in nomogram model 2**

| **Age** | points |
| --- | --- |
| ＜40 | 0 |
| 41-49 | 16 |
| 50-59 | 16 |
| 60-69 | 18 |
| 70+ | 4 |
|  |  |
| **Primary site** | points |
| Cervix Uteri | 56 |
| Corpus Uteri | 54 |
| Other Female Genital Organs | 75 |
| Ovary | 0 |
| Kidney or Renal Pelvis or Urinary Bladder | 22 |
| Vagina | 3 |
|  |  |
| **Tumor differentiated Grade** | points |
| Unknown | 0 |
| Well differentiated; Grade I | 16 |
| Moderately differentiated; Grade II | 16 |
| Poorly differentiated; Grade III | 18 |
| Undifferentiated; anaplastic; Grade IV | 3 |
|  |  |
| **SEER Stage** | points |
| Localized | 0 |
| Regional | 29 |
| Distant | 100 |
|  |  |
| **FIGO Stage** | points |
| FIGO I | 84 |
| FIGO II | 65 |
| FIGO III | 0 |
| FIGO IV | 10 |
|  |  |
|  |  |
| **Total Points** | **Probability for 3-year survival** |
| 195 | 0.1 |
| 192 | 0.2 |
| 190 | 0.3 |
| 188 | 0.4 |
| 186 | 0.5 |
| 184 | 0.6 |
| 181 | 0.7 |
| 178 | 0.8 |
| 172 | 0.9 |
|  |  |
| **Total Points** | **Probability for 5-year survival** |
| 191 | 0.1 |
| 188 | 0.2 |
| 186 | 0.3 |
| 184 | 0.4 |
| 182 | 0.5 |
| 179 | 0.6 |
| 177 | 0.7 |
| 173 | 0.8 |
| 168 | 0.9 |
|  |  |
| **Total Points** | **Probability for 8-year survival** |
| 188 | 0.1 |
| 185 | 0.2 |
| 183 | 0.3 |
| 181 | 0.4 |
| 179 | 0.5 |
| 177 | 0.6 |
| 174 | 0.7 |
| 171 | 0.8 |
| 165 | 0.9 |
