# Supplementary material for: Identification of characteristics and construction of nomogram to predict the survival probability of mesonephric carcinoma patients: A population‐based analysis and a case report
Source: Cancer Rep (Hoboken). 2023 Nov 29;7(1):e1940. doi: 10.1002/cnr2.1940 (PMC10809193; doi:10.1002/cnr2.1940)
Supplement: Supplementary file 3 — Table S1. Patient characteristics and clinicopathological variables with or without surgery performed [file CNR2-7-e1940-s004.doc]

**Supplementary Table 1．Patient characteristics and clinicopathological variables with or without surgery performed**

| **Variables** | **Total** | **Surgery** | | ***P* value** |
| --- | --- | --- | --- | --- |
|  |  | **Yes** | **No** |  |
| **N** | 65 | 61 | 4 |  |
| **Survival months** | 84.2±50.66 | 54.7 ± 13.0 | 57.5 ± 5.9 | 0.672 |
| **Age** | 54.83 ± 12.68 | 55.21 ± 12.65 | 54.09 ± 13.02 | 0.740 |
| **Age group** |  |  |  | 0.518 |
| ≤40 | 7 (10.77%) | 7 (11.5%) | 0 (0.0%) |  |
| 41-49 | 11 (16.92%) | 11 (18.0%) | 0 (0.0%) |  |
| 50-59 | 24 (36.92%) | 22 (36.1%) | 2 (50.0%) |  |
| 60-69 | 15 (23.08%) | 13 (21.3%) | 2 (50.0%) |  |
| 70+ | 8 (12.31%) | 8 (13.1%) | 0 (0.0%) |  |
| **Race** |  |  |  | 0.432 |
| White | 48 (73.85%) | 45 (73.8%) | 3 (75.0%) |  |
| Black | 6 (9.23%) | 5 (8.2%) | 1 (25.0%) |  |
| Other | 11 (16.92%) | 11 (18.0%) | 0 (0.0%) |  |
| **Primary site** |  |  |  | 0.921 |
| Cervix Uteri | 35 (53.85%) | 32 (52.5%) | 3 (75.0%) |  |
| Corpus Uteri | 14 (21.54%) | 13 (21.3%) | 1 (25.0%) |  |
| Other Female Genital Organs | 8 (12.31%) | 8 (13.1%) | 0 (0.0%) |  |
| Ovary | 3 (4.62%) | 3 (4.9%) | 0 (0.0%) |  |
| Kidney or Renal Pelvis or Urinary Bladder | 3 (4.62%) | 3 (4.9%) | 0 (0.0%) |  |
| Vagina | 2 (3.08%) | 2 (3.3%) | 0 (0.0%) |  |
| **Tumor differentiated grade** |  |  |  | <0.001* |
| Unknown | 26 (40.00%) | 25 (41.0%) | 1 (25.0%) |  |
| Well differentiated; Grade I | 14 (21.54%) | 14 (23.0%) | 0 (0.0%) |  |
| Moderately differentiated; Grade II | 16 (24.62%) | 16 (26.2%) | 0 (0.0%) |  |
| Poorly differentiated; Grade III | 7 (10.77%) | 4 (6.6%) | 3 (75.0%) |  |
| Undifferentiated; anaplastic; Grade IV | 2 (3.08%) | 2 (3.3%) | 0 (0.0%) |  |
| **SEER Stage** |  |  |  | 0.442 |
| Localized | 33 (50.77%) | 32 (52.5%) | 1 (25.0%) |  |
| Regional | 24 (36.92%) | 22 (36.1%) | 2 (50.0%) |  |
| Distant | 8 (12.31%) | 7 (11.5%) | 1 (25.0%) |  |
| **Duration from diagnosis to treatment** |  |  |  | 0.599 |
| less than 1 month | 43 (66.15%) | 41 (67.2%) | 2 (50.0%) |  |
| more than 1 month | 22 (33.85%) | 20 (32.8%) | 2 (50.0%) |  |
| **Chemotherapy** |  |  |  | 0.291 |
| No | 43 (66.15%) | 39 (63.9%) | 4 (100.0%) |  |
| Yes | 22 (33.85%) | 22 (36.1%) | 0 (0.0%) |  |
| **Radiotherapy** |  |  |  | 0.288 |
| No radiotherapy | 41 (63.08%) | 37 (60.7%) | 4 (100.0%) |  |
| Radotherapy prior or after surgery | 24 (36.92%) | 24 (39.3%) | 0 (0.0%) |  |
| **Lymph nodes resection** |  |  |  | 0.030* |
| No | 28 (43.08%) | 24 (39.3%) | 4 (100.0%) |  |
| Yes | 37 (56.92%) | 37 (60.7%) | 0 (0.0%) |  |
| **Regional LN examined** |  |  |  | 0.078 |
| None | 25 (38.46%) | 21 (34.4%) | 4 (100.0%) |  |
| ≤10 | 11 (16.92%) | 11 (18.0%) | 0 (0.0%) |  |
| 11 to 20 | 24 (36.92%) | 24 (39.3%) | 0 (0.0%) |  |
| ≥21 | 5 (7.69%) | 5 (8.2%) | 0 (0.0%) |  |
| **Regional LN positive** |  |  |  | 0.033* |
| 0 | 35 (53.85%) | 35 (57.4%) | 0 (0.0%) |  |
| more than 1 | 5 (7.69%) | 5 (8.2%) | 0 (0.0%) |  |
| No LN examined | 25 (38.46%) | 21 (34.4%) | 4 (100.0%) |  |
| **Bone Metastasis** |  |  |  | 0.796 |
| No | 64 (98.46%) | 60 (98.4%) | 4 (100.0%) |  |
| Yes | 1 (1.54%) | 1 (1.6%) | 0 (0.0%) |  |
| **Lung Metastasis** |  |  |  | 0.120 |
| No | 63 (96.92%) | 60 (98.4%) | 3 (75.0%) |  |
| Yes | 2 (3.08%) | 1 (1.6%) | 1 (25.0%) |  |
| **Tumor Size** |  |  |  | 0.004* |
| ≤5 cm | 36 (55.38%) | 36 (59.0%) | 0 (0.0%) |  |
| ＞5 cm | 11 (16.92%) | 8 (13.1%) | 3 (75.0%) |  |
| Unknown | 18 (27.69%) | 17 (27.9%) | 1 (25.0%) |  |
| **FIGO stage** |  |  |  | 0.002* |
| FIGO I | 37 (56.92%) | 36 (59.0%) | 1 (25.0%) |  |
| FIGO II | 17 (26.15%) | 16 (26.2%) | 1 (25.0%) |  |
| FIGO III | 7 (10.77%) | 6 (9.8%) | 1 (25.0%) |  |
| FIGO IV | 3 (4.62%) | 3 (4.9%) | 0 (0.0%) |  |
| Unknown | 1 (1.54%) | 0 (0.0%) | 1 (25.0%) |  |
| **Cancer specific dead** |  |  |  | 0.226 |
| Alive | 50 (76.92%) | 48 (78.7%) | 2 (50.0%) |  |
| Dead | 15 (23.08%) | 13 (21.3%) | 2 (50.0%) |  |
| **Cancer competitive dead** |  |  |  | 0.003* |
| Alive | 44 (67.69%) | 44 (72.1%) | 0 (0.0%) |  |
| Die for cancer | 15 (23.08%) | 13 (21.3%) | 2 (50.0%) |  |
| Die for other cause | 6 (9.23%) | 4 (6.6%) | 2 (50.0%) |  |

^*^Statistically significant (*P* < 0.05)
