# Supplementary material for: Identification of characteristics and construction of nomogram to predict the survival probability of mesonephric carcinoma patients: A population‐based analysis and a case report
Source: Cancer Rep (Hoboken). 2023 Nov 29;7(1):e1940. doi: 10.1002/cnr2.1940 (PMC10809193; doi:10.1002/cnr2.1940)
Supplement: Supplementary file 4 — Table S2. Patient characteristics and clinicopathological variables with or without radiotherapy performed [file CNR2-7-e1940-s003.doc]

**Supplementary Table 2．Patient characteristics and clinicopathological variables with or without radiotherapy performed**

| **Variables** | **Total** | ***Radiotherapy*** | | ***P* value** |
| --- | --- | --- | --- | --- |
|  |  | ***No*** | **Yes** |  |
| **N** | 65 | 41 | 24 |  |
| **Survival months** | 84.2±50.66 | 75.8 ± 51.9 | 98.7 ± 46.0 | 0.064 |
| **Age** | 54.83 ± 12.68 | 53.5 ± 13.8 | 57.1 ± 10.4 | 0.554 |
| **Age group** |  |  |  | 0.163 |
| ≤40 | 7 (10.77%) | 7 (17.1%) | 0 (0.0%) |  |
| 41-49 | 11 (16.92%) | 6 (14.6%) | 5 (20.8%) |  |
| 50-59 | 24 (36.92%) | 13 (31.7%) | 11 (45.8%) |  |
| 60-69 | 15 (23.08%) | 11 (26.8%) | 4 (16.7%) |  |
| 70+ | 8 (12.31%) | 4 (9.8%) | 4 (16.7%) |  |
| **Race** |  |  |  | 0.109 |
| White | 48 (73.85%) | 28 (68.3%) | 20 (83.3%) |  |
| Black | 6 (9.23%) | 3 (7.3%) | 3 (12.5%) |  |
| Other | 11 (16.92%) | 10 (24.4%) | 1 (4.2%) |  |
| **Primary site** |  |  |  | 0.039* |
| Cervix Uteri | 35 (53.85%) | 16 (39.0%) | 19 (79.2%) |  |
| Corpus Uteri | 14 (21.54%) | 11 (26.8%) | 3 (12.5%) |  |
| Other Female Genital Organs | 8 (12.31%) | 7 (17.1%) | 1 (4.2%) |  |
| Ovary | 3 (4.62%) | 3 (7.3%) | 0 (0.0%) |  |
| Kidney or Renal Pelvis or Urinary Bladder | 3 (4.62%) | 3 (7.3%) | 0 (0.0%) |  |
| Vagina | 2 (3.08%) | 1 (2.4%) | 1 (4.2%) |  |
| **Tumor differentiated grade** |  |  |  | 0.337 |
| Unknown | 26 (40.00%) | 20 (48.8%) | 6 (25.0%) |  |
| Well differentiated; Grade I | 14 (21.54%) | 7 (17.1%) | 7 (29.2%) |  |
| Moderately differentiated; Grade II | 16 (24.62%) | 10 (24.4%) | 6 (25.0%) |  |
| Poorly differentiated; Grade III | 7 (10.77%) | 3 (7.3%) | 4 (16.7%) |  |
| Undifferentiated; anaplastic; Grade IV | 2 (3.08%) | 1 (2.4%) | 1 (4.2%) |  |
| **SEER Stage** |  |  |  | 0.769 |
| Localized | 33 (50.77%) | 21 (51.2%) | 12 (50.0%) |  |
| Regional | 24 (36.92%) | 14 (34.1%) | 10 (41.7%) |  |
| Distant | 8 (12.31%) | 6 (14.6%) | 2 (8.3%) |  |
| **Duration from diagnosis to treatment** |  |  |  | 0.947 |
| less than 1 month | 43 (66.15%) | 27 (65.9%) | 16 (66.7%) |  |
| more than 1 month | 22 (33.85%) | 14 (34.1%) | 8 (33.3%) |  |
| **Surgery** |  |  |  | 0.288 |
| Surgery performed | 61 (93.85%) | 37 (90.2%) | 24 (100.0%) |  |
| Not recommended | 4 (6.15%) | 4 (9.8%) | 0 (0.0%) |  |
| **Chemotherapy** |  |  |  | 0.008* |
| No | 43 (66.15%) | 32 (78.0%) | 11 (45.8%) |  |
| Yes | 22 (33.85%) | 9 (22.0%) | 13 (54.2%) |  |
| **Lymph nodes resection** |  |  |  | 0.731 |
| No | 28 (43.08%) | 17 (41.5%) | 11 (45.8%) |  |
| Yes | 37 (56.92%) | 24 (58.5%) | 13 (54.2%) |  |
| **Regional LN examined** |  |  |  | 0.836 |
| None | 25 (38.46%) | 16 (39.0%) | 9 (37.5%) |  |
| ≤10 | 11 (16.92%) | 7 (17.1%) | 4 (16.7%) |  |
| 11 to 20 | 24 (36.92%) | 14 (34.1%) | 10 (41.7%) |  |
| ≥21 | 5 (7.69%) | 4 (9.8%) | 1 (4.2%) |  |
| **Regional LN positive** |  |  |  | 0.985 |
| 0 | 35 (53.85%) | 22 (53.7%) | 13 (54.2%) |  |
| more than 1 | 5 (7.69%) | 3 (7.3%) | 2 (8.3%) |  |
| No LN examined | 25 (38.46%) | 16 (39.0%) | 9 (37.5%) |  |
| **Bone Metastasis** |  |  |  | 0.369 |
| No | 64 (98.46%) | 41 (100.0%) | 23 (95.8%) |  |
| Yes | 1 (1.54%) | 0 (0.0%) | 1 (4.2%) |  |
| **Lung Metastasis** |  |  |  | 0.527 |
| No | 63 (96.92%) | 39 (95.1%) | 24 (100.0%) |  |
| Yes | 2 (3.08%) | 2 (4.9%) | 0 (0.0%) |  |
| **Tumor Size** |  |  |  | 0.067 |
| ≤5 cm | 36 (55.38%) | 19 (46.3%) | 17 (70.8%) |  |
| ＞5 cm | 11 (16.92%) | 10 (24.4%) | 1 (4.2%) |  |
| Unknown | 18 (27.69%) | 12 (29.3%) | 6 (25.0%) |  |
| **FIGO stage** |  |  |  | 0.507 |
| FIGO I | 37 (56.92%) | 23 (56.1%) | 14 (58.3%) |  |
| FIGO II | 17 (26.15%) | 9 (22.0%) | 8 (33.3%) |  |
| FIGO III | 7 (10.77%) | 5 (12.2%) | 2 (8.3%) |  |
| FIGO IV | 3 (4.62%) | 3 (7.3%) | 0 (0.0%) |  |
| Unknown | 1 (1.54%) | 1 (2.4%) | 0 (0.0%) |  |
| **Cancer specific dead** |  |  |  | 0.122 |
| Alive | 50 (76.92%) | 29 (70.7%) | 21 (87.5%) |  |
| Dead | 15 (23.08%) | 12 (29.3%) | 3 (12.5%) |  |
| **Cancer competitive dead** |  |  |  | 0.270 |
| Alive | 44 (67.69%) | 25 (61.0%) | 19 (79.2%) |  |
| Die for cancer | 15 (23.08%) | 12 (29.3%) | 3 (12.5%) |  |
| Die for other cause | 6 (9.23%) | 4 (9.8%) | 2 (8.3%) |  |

^*^Statistically significant (*P* < 0.05)
