# Supplementary material for: Identification of characteristics and construction of nomogram to predict the survival probability of mesonephric carcinoma patients: A population‐based analysis and a case report
Source: Cancer Rep (Hoboken). 2023 Nov 29;7(1):e1940. doi: 10.1002/cnr2.1940 (PMC10809193; doi:10.1002/cnr2.1940)
Supplement: Supplementary file 5 — Table S3. Patient characteristics and clinicopathological variables with or without chemotherapy performed [file CNR2-7-e1940-s005.doc]

**Supplementary Table 3．Patient characteristics and clinicopathological variables with or without chemotherapy performed**

| **Variables** | **Total** | ***Chemotherapy*** | | ***P* value** |
| --- | --- | --- | --- | --- |
|  |  | ***No*** | **Yes** |  |
| **N** | 65 | 43 | 22 |  |
| **Survival months** | 84.2±50.66 | 84.51 ± 50.33 | 83.64 ± 52.47 | 0.873 |
| **Age** | 54.83 ± 12.68 | 55.21 ± 12.65 | 54.09 ± 13.02 | 0.740 |
| **Age group** |  |  |  | 0.371 |
| ≤40 | 7 (10.77%) | 5 (11.63%) | 2 (9.09%) |  |
| 41-49 | 11 (16.92%) | 6 (13.95%) | 5 (22.73%) |  |
| 50-59 | 24 (36.92%) | 14 (32.56%) | 10 (45.45%) |  |
| 60-69 | 15 (23.08%) | 13 (30.23%) | 2 (9.09%) |  |
| 70+ | 8 (12.31%) | 5 (11.63%) | 3 (13.64%) |  |
| **Race** |  |  |  | 0.981 |
| White | 48 (73.85%) | 32 (74.42%) | 16 (72.73%) |  |
| Black | 6 (9.23%) | 4 (9.30%) | 2 (9.09%) |  |
| Other | 11 (16.92%) | 7 (16.28%) | 4 (18.18%) |  |
| **Primary site** |  |  |  | 0.731 |
| Cervix Uteri | 35 (53.85%) | 22 (51.16%) | 13 (59.09%) |  |
| Corpus Uteri | 14 (21.54%) | 9 (20.93%) | 5 (22.73%) |  |
| Other Female Genital Organs | 8 (12.31%) | 5 (11.63%) | 3 (13.64%) |  |
| Ovary | 3 (4.62%) | 2 (4.65%) | 1 (4.55%) |  |
| Kidney or Renal Pelvis or Urinary Bladder | 3 (4.62%) | 3 (6.98%) | 0 (0.00%) |  |
| Vagina | 2 (3.08%) | 2 (4.65%) | 0 (0.00%) |  |
| **Tumor differentiated grade** |  |  |  | 0.757 |
| Unknown | 26 (40.00%) | 15 (34.88%) | 11 (50.00%) |  |
| Well differentiated; Grade I | 14 (21.54%) | 10 (23.26%) | 4 (18.18%) |  |
| Moderately differentiated; Grade II | 16 (24.62%) | 12 (27.91%) | 4 (18.18%) |  |
| Poorly differentiated; Grade III | 7 (10.77%) | 5 (11.63%) | 2 (9.09%) |  |
| Undifferentiated; anaplastic; Grade IV | 2 (3.08%) | 1 (2.33%) | 1 (4.55%) |  |
| **SEER Stage** |  |  |  | 0.004* |
| Localized | 33 (50.77%) | 25 (58.14%) | 8 (36.36%) |  |
| Regional | 24 (36.92%) | 17 (39.53%) | 7 (31.82%) |  |
| Distant | 8 (12.31%) | 1 (2.33%) | 7 (31.82%) |  |
| **Duration from diagnosis to treatment** |  |  |  | 0.805 |
| less than 1 month | 43 (66.15%) | 28 (65.12%) | 15 (68.18%) |  |
| more than 1 month | 22 (33.85%) | 15 (34.88%) | 7 (31.82%) |  |
| **Surgery** |  |  |  |  |
| Surgery performed | 61 (93.85%) | 39 (90.70%) | 22 (100.00%) | 0.291 |
| Not recommended | 4 (6.15%) | 4 (9.30%) | 0 (0.00%) |  |
| **Radiotherapy** |  |  |  | 0.008* |
| No radiotherapy | 41 (63.08%) | 32 (74.42%) | 9 (40.91%) |  |
| Radotherapy prior or after surgery | 24 (36.92%) | 11 (25.58%) | 13 (59.09%) |  |
| **Lymph nodes resection** |  |  |  | 0.782 |
| No | 28 (43.08%) | 18 (41.86%) | 10 (45.45%) |  |
| Yes | 37 (56.92%) | 25 (58.14%) | 12 (54.55%) |  |
| **Regional LN examined** |  |  |  | 0.576 |
| None | 25 (38.46%) | 17 (39.53%) | 8 (36.36%) |  |
| ≤10 | 11 (16.92%) | 9 (20.93%) | 2 (9.09%) |  |
| 11 to 20 | 24 (36.92%) | 14 (32.56%) | 10 (45.45%) |  |
| ≥21 | 5 (7.69%) | 3 (6.98%) | 2 (9.09%) |  |
| **Regional LN positive** |  |  |  | 0.080 |
| 0 | 35 (53.85%) | 25 (58.14%) | 10 (45.45%) |  |
| more than 1 | 5 (7.69%) | 1 (2.33%) | 4 (18.18%) |  |
| No LN examined | 25 (38.46%) | 17 (39.53%) | 8 (36.36%) |  |
| **Bone Metastasis** |  |  |  | 0.338 |
| No | 64 (98.46%) | 43 (100.00%) | 21 (95.45%) |  |
| Yes | 1 (1.54%) | 0 (0.00%) | 1 (4.55%) |  |
| **Lung Metastasis** |  |  |  | 1.000 |
| No | 63 (96.92%) | 42 (97.67%) | 21 (95.45%) |  |
| Yes | 2 (3.08%) | 1 (2.33%) | 1 (4.55%) |  |
| **Tumor Size** |  |  |  | 0.244 |
| ≤5 cm | 36 (55.38%) | 27 (62.79%) | 9 (40.91%) |  |
| ＞5 cm | 11 (16.92%) | 6 (13.95%) | 5 (22.73%) |  |
| Unknown | 18 (27.69%) | 10 (23.26%) | 8 (36.36%) |  |
| **FIGO stage** |  |  |  | 0.061 |
| FIGO I | 37 (56.92%) | 27 (62.79%) | 10 (45.45%) |  |
| FIGO II | 17 (26.15%) | 12 (27.91%) | 5 (22.73%) |  |
| FIGO III | 7 (10.77%) | 3 (6.98%) | 4 (18.18%) |  |
| FIGO IV | 3 (4.62%) | 0 (0.00%) | 3 (13.64%) |  |
| Unknown | 1 (1.54%) | 1 (2.33%) | 0 (0.00%) |  |
| **Cancer specific dead** |  |  |  | 0.503 |
| Alive | 50 (76.92%) | 32 (74.42%) | 18 (81.82%) |  |
| Dead | 15 (23.08%) | 11 (25.58%) | 4 (18.18%) |  |
| **Cancer competitive dead** |  |  |  | 0.844 |
| Alive | 44 (67.69%) | 28 (65.12%) | 16 (72.73%) |  |
| Die for cancer | 15 (23.08%) | 11 (25.58%) | 4 (18.18%) |  |
| Die for other cause | 6 (9.23%) | 4 (9.30%) | 2 (9.09%) |  |

^*^Statistically significant (*P* < 0.05)
